# Nonenzymatic Functions of Acetylcholinesterase Splice Variants in the Developmental Neurotoxicity of Organophosphates: Chlorpyrifos, Chlorpyrifos Oxon, and Diazinon

**DOI:** 10.1289/ehp.9487

**Published:** 2006-10-11

**Authors:** Ruth R. Jameson, Frederic J. Seidler, Theodore A. Slotkin

**Affiliations:** Department of Pharmacology & Cancer Biology, Duke University Medical Center, Durham, North Carolina, USA

**Keywords:** acetylcholinesterase, brain development, chlorpyrifos, diazinon, organophosphate insecticides, PC12 cells

## Abstract

**Background:**

Organophosphate pesticides affect mammalian brain development through mechanisms separable from the inhibition of acetylcholinesterase (AChE) enzymatic activity and resultant cholinergic hyperstimulation. In the brain, AChE has two catalytically similar splice variants with distinct functions in development and repair. The rare, read-through isoform, AChE-R, is preferentially induced by injury and appears to promote repair and protect against neurodegeneration. Overexpression of the more abundant, synaptic isoform, AChE-S, enhances neurotoxicity.

**Objectives:**

We exposed differentiating PC12 cells, a model for developing neurons, to 30 μM chlorpyrifos (CPF) or diazinon (DZN), or CPF oxon, the active metabolite that irreversibly inhibits AChE enzymatic activity, in order to determine whether they differentially induce the formation of AChE-S as a mechanistic predictor of developmental neurotoxicity. We then administered CPF or DZN to neonatal rats on postnatal days 1–4 using daily doses spanning the threshold for AChE inhibition (0–20%); we then evaluated AChE gene expression in forebrain and brainstem on post-natal day 5.

**Results:**

In PC12 cells, after 48 hr of exposure, CPF, CPF oxon, and DZN enhanced gene expression for AChE-R by about 20%, whereas CPF and DZN, but not CPF oxon, increased AChE-S expression by 20–40%. Thus, despite the fact that CPF oxon is a much more potent AChE inhibitor, it is the native compound (CPF) that induces expression of the neurotoxic AChE-S iso-form. For *in vivo* exposures, 1 mg/kg CPF had little or no effect, but 0.5 or 2 mg/kg DZN induced both AChE-R and AChE-S, with a greater effect in males.

**Conclusions:**

Our results indicate that nonenzymatic functions of AChE variants may participate in and be predictive of the relative developmental neurotoxicity of organophosphates, and that the various organophosphates differ in the degree to which they activate this mechanism.

The household use of some of the organophosphate (OP) insecticides has been restricted in recent years after findings of neurodevelopmental toxicity and unpredicted environmental persistence [U.S. Environmental Protection Agency (EPA) 2000, 2002]. In spite of these constraints, chlorpyrifos (CPF) and diazinon (DZN) each continue to be applied in agriculture at rates > 10 million pounds annually (U.S. EPA 2004, 2006). In animal models, developmental neurotoxicity results from either CPF or DZN at doses that do not elicit any signs of systemic intoxication and even at exposures below the threshold for inhibition of acetylcholinesterase (AChE), which is often considered to be the primary target for OPs (Slotkin 1999, 2004, 2005; Slotkin et al. 2006a, 2006b). Because pregnant women are likely to be exposed to OPs under circumstances that do not elicit outward signs of intoxication (De Peyster et al. 1993; Gurunathan et al. 1998; Ostrea et al. 2002) and in light of recent findings that such exposures can produce long-term cognitive impairment in their children (Rauh et al. 2006; Rohlman et al. 2005), the mechanisms and consequences of OP-induced developmental neurotoxicity remain a major environmental concern.

The systemic toxicity of OPs reflects the symptoms related to cholinergic hyper-stimulation consequent to the irreversible loss of AChE catalytic activity, which typically emerges when inhibition exceeds 70% (Clegg and van Gemert 1999). In contrast, CPF and DZN both elicit adverse effects on brain development at much lower exposures, and the adverse effects involve the native compounds and not their hepatically produced oxon metabolites, which are the actual agents that cause irreversible AChE inhibition (Slotkin 1999, 2004, 2005; Slotkin et al. 2006a, 2006b). Nevertheless, AChE itself may be involved in these effects through mechanisms unrelated to its catalytic activity and therefore separable from “cholinergic” effects. As a structural protein, AChE appears to play an important role in axonal outgrowth (Bigbee et al. 2000), synaptogenesis (Sternfeld et al. 1998), cell adhesion (Bigbee and Sharma 2004), and neuronal migration (Byers et al. 2005; Dori et al. 2005), functions that are largely independent of the enzymatic ability to hydrolyze ACh (Soreq and Seidman 2001). It is critical to note that these nonenzymatic functions differ among the AChE isoforms and/or their cleavage products (Dori et al. 2005; Grifman et al. 1998; Sternfeld et al. 1998). The open reading frame of the AChE gene comprises five exons, of which exons 2, 3, and 4 contain the catalytic subunits and are invariantly spliced (Figure 1). Alternate splicing of the last two exons and their associated introns results in at least three variants of the mRNA, two of which are expressed in the central nervous system. The most abundant “synaptic” isoform (AChE-S) is typically tetrameric and membrane-bound in the synapse, where it performs the bulk of ACh hydrolysis, whereas as a much rarer “read-through” variant (AChE-R)—so called because it results from inclusion of intron 4—is soluble and monomeric. In general, although both AChE isoforms are inducible by cholinesterase inhibitors or by neural injury, the expression of AChE-R appears to be related to neuroprotection and repair, whereas AChE-S, either by itself or when up-regulated in conjunction with AChE-R, is more highly associated with injury and increasing neurotoxicity (Cohen et al. 2002; Perrier et al. 2005; Shohami et al. 2000; Sternfeld et al. 2000). In this context, over-expression of AChE-S produces aberrant dendritic morphology and neuronal fragmentation (Sternfeld et al. 2000). Accordingly, recent studies have explored the roles of the two AChE variants in the response to neuronal injury and neurodegeneration (Perrier et al. 2005; Sternfeld et al. 2000), physical trauma (Shohami et al. 2000), and Alzheimer disease (Darreh-Shori et al. 2004).

Cholinesterase inhibitors, including the OPs, are known to increase the overall expression of AChE and to alter the relative expression of AChE-R and AChE-S in the adult brain (Damodaran et al. 2003; Darreh-Shori et al. 2004; Friedman et al. 1996; Meshorer et al. 2002; Salmon et al. 2005). In the present study, we explored the corresponding effects in the developing nervous system to evaluate the potential contribution of nonenzymatic function of AChE splice variants as a target for the developmental neurotoxicity of OPs. First, we made use of PC12 cells, an *in vitro* model of neuronal development that has proven especially useful for evaluations of developmental neurotoxicants, including the OPs (Crumpton et al. 2000; Das and Barone 1999; Fujita et al. 1989; Jameson et al. 2006; Madhok and Sharp 1992; Qiao et al. 2001, 2005; Song et al. 1998; Teng and Greene 1994). Upon addition of nerve growth factor (NGF), PC12 cells begin to differentiate and develop axonal projections and electrical excitability, along with the properties of cholinergic neurons, including increased expression of AChE (Das and Barone 1999; Fujita et al. 1989; Greene and Rukenstein 1981; Teng and Greene 1994). In this model, we compared the effects of CPF and DZN to that of CPF oxon (CPO), which is three orders of magnitude more potent toward inhibition of AChE than the parent compound, CPF (Das and Barone 1999). Next, we compared CPF to DZN in developing rats given treatment with either agent on postnatal days (PND) 1–4, using doses spanning the threshold for AChE inhibition that are known to evoke neurodevelopmental deficits without signs of systemic toxicity (Slotkin 1999, 2004, 2005; Slotkin et al. 2006a, 2006b). Because the two AChE isoforms share the common catalytic domain and therefore hydrolyze acetylcholine with similar efficiency (Sternfeld et al. 1998), enzymatic techniques cannot distinguish AChE-R from AChE-S, so instead, we evaluated the differential effects on expression of mRNAs encoding the two variants, using standard reverse transcriptase-polymerase chain reaction (RT-PCR) methods.

## Methods

### PC12 cell cultures

Because of the clonal instability of the PC12 cell line (Fujita et al. 1989), the experiments were performed on cells that had undergone fewer than five passages, and all experiments were repeated with multiple batches of cells. As described previously (Crumpton et al. 2000; Qiao et al. 2003; Song et al. 1998), PC12 cells (American Type Culture Collection, 1721-CRL; obtained from the Duke University Comprehensive Cancer Center, Durham, NC) were seeded onto 100-mm poly-d-lysine–coated plates (approximately 3 × 106 cells/plate) in RPMI-1640 medium (Invitrogen, Carlsbad, CA) supplemented with 10% inactivated horse serum (Sigma Chemical Co., St. Louis, MO), 5% fetal bovine serum (Sigma Chemical Co.), and 50 μg/mL penicillin streptomycin (Invitrogen); cells were incubated with 7.5% CO_2_ at 37°C. Twenty-four hours after seeding, the medium was changed to include 50 ng/mL 2.5 S murine NGF (Invitrogen), along with CPF, CPO, or DZN (Chem Service, West Chester, PA). The OP agents were dissolved in dimethylsulfoxide (DMSO; Sigma Chemical Co.), achieving a final DMSO concentration of 0.1% in the culture medium, and the corresponding control samples contained equivalent DMSO concentrations. This concentration of DMSO has no effect on PC12 cells (Qiao et al. 2001, 2003; Song et al. 1998). Samples were then incubated for 48 hr in the continuous presence of each agent and NGF. We evaluated the effects of 30 μM CPF, CPO, or DZN, a concentration known to produce adverse effects on cell replication and differentiation (Crumpton et al. 2000; Jameson et al. 2006; Qiao et al. 2001; Song et al. 1998); in addition, we evaluated a 1,000× lower concentration of CPO (30 nM) in light of its correspondingly higher potency as an AChE inhibitor (Das and Barone 1999).

### Animal treatments

All animal experiments were approved by the Duke University Animal Care and Use Committee and were carried out in accordance with all federal and state standards of care; animals were treated humanely and with regard for alleviation of suffering. Timed-pregnant Sprague-Dawley rats (Charles River, Raleigh, NC) were housed in breeding cages, with a 12-hr light/dark cycle and free access to food and water. On the day of birth, all pups were randomized and redistributed to the dams with a litter size of 9–10 to maintain a standard nutritional status.

CPF and DZN were dissolved in DMSO to provide consistent absorption (Whitney et al. 1995) and were injected subcutaneously in a volume of 1 mL/kg once daily on PND1–4; control animals received equivalent injections of the DMSO vehicle. For both agents, we used doses below the threshold for growth retardation and systemic toxicity (Campbell et al. 1997; Slotkin et al. 2006a; Whitney et al. 1995): 1 mg/kg CPF and 0.5 or 2 mg/kg DZN. This CPF treatment and the higher dose of DZN produce neurotoxicity in developing rat brain while eliciting < 20% AChE inhibition, well below the 70% threshold necessary for symptoms of cholinergic hyperstimulation (Clegg and van Gemert 1999); the lower dose of DZN is below the threshold for detectable AChE inhibition (Slotkin 1999, 2004; Slotkin et al. 2006b; Song et al. 1997; Whitney et al. 1995). These treatments thus resemble the nonsymptomatic exposures reported in pregnant women (De Peyster et al. 1993) and are within the range of expected fetal and childhood exposures after routine home application or in agricultural communities (Gurunathan et al. 1998; Ostrea et al. 2002). On PND5 (24 hr after the last dose), one male and one female pup were selected from each of six litters in each treatment group and were used for evaluations. Animals were decapitated, the cerebellum (which is sparse in acetylcholine projections) was removed, and the rest of the brain was separated into brainstem and forebrain (regions containing the majority of acetylcholine projections) by a cut made rostral to the thalamus. Tissues were weighed and flash-frozen in liquid nitrogen and maintained at –45°C until analyzed. None of the treatments led to changes in weight of body or brain region, and there was no loss of viability.

### AChE variant analysis by RT-PCR

Total RNA was extracted using the Aurum Total RNA Fatty and Fibrous Tissue Kit (Bio-Rad Laboratories, Hercules, CA), following the manufacturer’s protocol, yielding approximately 1 μg total RNA per milligram tissue. We verified the integrity of the RNA by ethidium bromide staining of the ribosomal bands isolated by standard electrophoresis in agarose, and determined the concentration and purity. PCR amplification was performed using standard commercial reagent kits (Invitrogen). A 5-μg aliquot of each RNA sample was reverse-transcribed with Superscript III using random hexamer primers. The isoforms were amplified in separate PCR reactions using primers designed for each specific splice variant (Table 1). Because the expression of cytoskeletal and metabolic genes changes during neuronal development, the typical, constitutively expressed mRNAs (“housekeeping genes”) were inappropriate for use as internal standards. Instead, we used QuantumRNA Classic 18S Internal Standard Primers (Ambion Inc., Austin, TX), which amplify a portion of the 18S ribosomal subunit. This standard can be titrated to generate a consistent PCR signal across different numbers of cycles by altering the ratio of 18S primers and competimers, the latter of which inhibit primer binding to the recognition sequence. The mRNA encoding the AChE-R variant is present in very low concentrations relative to that for AChE-S or other mRNAs (Perrier et al. 2005). Accordingly, we used different primer:competimer ratios for the two splice variants: 1:19 for AChE-R, and 1:4 for AChE-S. PCR reaction parameters were optimized such that both the AChE and 18S sequences were amplified in the linear range. The annealing temperature used for both isoforms was 55°C.

PCR products were quantified by electrophoresis of 10 μL of product on 1.5% agarose gels using Certified PCR Agarose (BioRad) containing 0.3 μg/mL ethidium bromide. Gel images were digitized and bands were quantified using NIH Image Software (National Institutes of Health 2006). Images were uniformly calibrated to an optical density step tablet, and the background was reduced with a rolling ball radius of 50. AChE band values for each isoform had the background subtracted and were then normalized to the corresponding 18S band for each sample.

### Data analysis

Data are presented as means and SEs, and treatment effects were evaluated by multivariate analysis of variance (ANOVA) incorporating all relevant factors: for the *in vitro* studies, treatment (control, CPF 30 μM, DZN 30 μM, CPO 30 μM, CPO 30 nM) and AChE subtype (AChE-R, AChE-S); for the *in vivo* studies, treatment (control, CPF 1 mg/kg, DZN 0.5 mg/kg, DZN 2 mg/kg), brain region (brainstem, forebrain), sex (male, female), and subtype (AChE-R, AChE-S). Lower-order tests were conducted as permitted by the interactions of treatment with other variables; however, where treatment did not interact with another variable, only main treatment effects are reported without further subdivision. For convenience, results are shown as the percent change from corresponding control values, but statistical evaluations were always conducted on the original data (log-transformed whenever the variance between groups was heterogeneous). Because each set of primers has distinct binding properties and requires different cycle parameters, values for different treatments are comparable for each splice variant alone, but comparing absolute values of the two variants to each other is not meaningful. Significance was assumed at *p* < 0.05.

## Results

**Figure 2:**
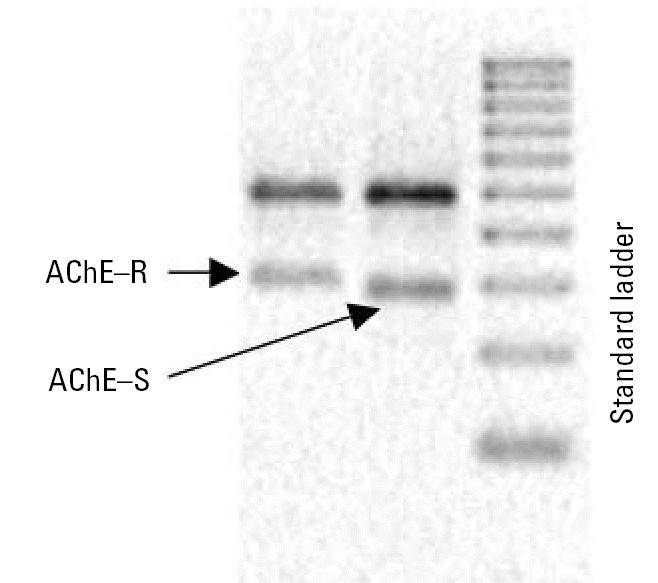
Sample gel lanes from a representative PC12 cell determination of AChE-R, AChE-S, and a DNA standard ladder. The upper band in the sample lanes is the 18S ribosomal control; the standard DNA ladder consists of increments of 100 base pairs.

The RT-PCR strategy yielded single products of the anticipated molecular weights for both of the AChE isoforms and for the ribosomal 18S internal standards (Figure 2). Amplification of the AChE-R splice variant required at least five cycles more than were necessary for detection of the AChE-S variant, consistent with its much lower abundance, even under conditions that increase expression (Perrier et al. 2005).

### In vitro *studies.*

**Figure 3:**
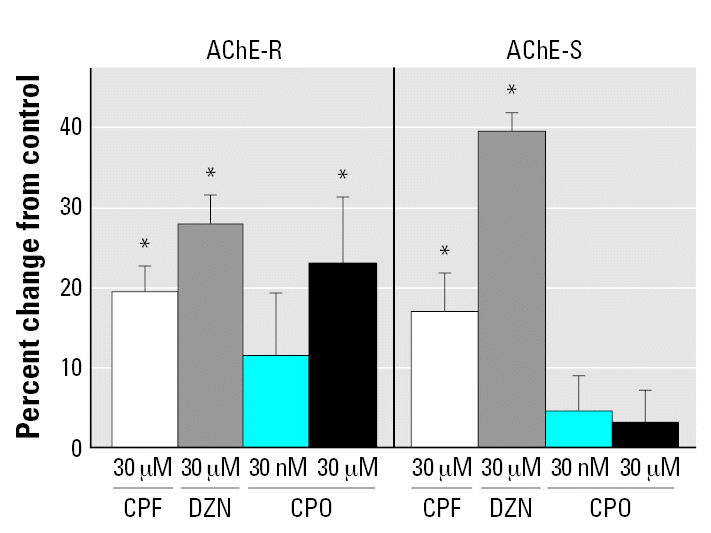
Effects of organophosphate treatment (CPF, DZN, CPO) on AChE mRNA splice variants in differentiating PC12 cells. Cells were treated for 48 hr in the presence of NGF. Data represent means and SEs obtained from 6–12 cultures for each condition, presented as the percent change from control values. Across both variants, ANOVA indicates a significant main treatment effect (*p* < 0.0001) and a difference in treatment effects between the two variants (treatment × subtype interaction, *p* < 0.0005). For each variant, there was a main treatment effect (*p* < 0.002 for AChE-R and *p* < 0.0001 for AChE-S). *Differs significantly from the corresponding control.

In control PC12 cells, expression values for the two AChE variants (ratio to 18S ribosomal RNA; see “Methods”) were 0.42 ± 0.02 for AChE-R and 0.51 ± 0.01 for AChE-S. As expected from earlier *in vivo* work with nerve gas OPs (Damodaran et al. 2003; Soreq and Seidman 2001), treatment of differentiating PC12 cells with CPF, DZN, or CPO evoked increases in AChE mRNA (Figure 3). At a CPF concentration of 30 μM, the mRNAs encoding both AChE-R and AChE-S exhibited increases of about 20%, whereas for DZN, the same concentration elicited greater induction (30% and 40%, respectively). Because CPO is approximately 1,000 times more potent than CPF as an AChE inhibitor (Das and Barone 1999), we first examined a concentration of 30 nM and found a smaller effect on AChE-R expression, intermediate between control levels and the higher values evoked by 30 μM CPF or DZN. Increasing the CPO concentration to 30 μM produced an effect similar to that of equimolar concentrations of CPF or DZN. Strikingly, CPO was totally ineffective in increasing AChE-S, even at the higher concentration.

### In vivo *studies.*

In control rats, the values (ratio to 18S ribosomal RNA; see “Methods”) for expression of the AChE subtypes were as follows: for AChE-R, 1.83 ± 0.12 in male brainstem, 1.67 ± 0.29 in female brainstem, 1.38 ± 0.05 in male forebrain, and 1.65 ± 0.26 in female forebrain; for AChE-S, 0.77 ± 0.03, 0.94 ± 0.03, 0.35 ± 0.02, and 0.65 ± 0.02, respectively. Whereas there was no statistically significant sex difference for AChE-R, the difference for AChE-S was highly significant: *p* < 0.0001 for the main effect of sex and *p* < 0.0001 for the interaction of sex × region, with the main effect of sex significant both for brainstem (*p* < 0.004) and forebrain (*p* < 0.0001).

**Figure 4:**
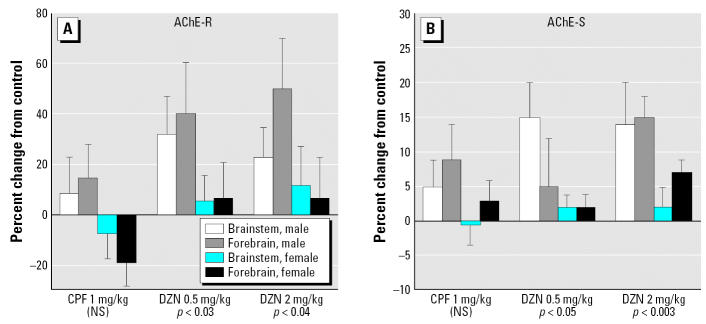
Effects of *in vivo* organophosphate treatment on AChE mRNA splice variants. (*A*) AChE-R. (*B*) AChE-S. Animals were treated with CPF or DZN at the indicated doses on PND1–4, and samples were obtained on PND5. Data represent mean and SE obtained from six animals of each sex in each treatment group, presented as the percent change from the corresponding control values. Across both variants, ANOVA indicates a significant main treatment effect (*p* < 0.005), which was also significant for each variant separately (*p* < 0.03 for AChE-R; *p* < 0.02 for AChE-S). Differences in males were statistically significant (*p* < 0.006), whereas those in females were not. Statistics for individual treatments whose main effects differ from the corresponding control values are shown at the bottom of each panel; tests for individual regions were not conducted because of the absence of treatment × region interactions. Note the difference in scales in (*A*) and (*B*). NS, not significant.

Daily treatment of neonatal rats with 1 mg/kg CPF evoked slight increments in AChE-R expression in brainstem and fore-brain of male rats (Figure 4A), an effect that did not achieve statistical significance (no significant main treatment effect or interaction of treatment × region). In contrast, DZN, either at 0.5 or 2 mg/kg, had more robust effects, producing significant increments (*p* < 0.03 and *p* < 0.04 respectively for main treatment effects), with preferentially greater effects in males (*p* < 0.02 for the main treatment effect) than in females (not significant). Similarly, for AChE-S (Figure 4B), DZN evoked significant increases in expression at either 0.5 or 2 mg/kg (*p* < 0.05 and *p* < 0.003 for main treatment effects), but the effects for CPF were insufficient to achieve statistical significance; again, the effects of DZN in males (*p* < 0.05 for the main treatment effect) exceeded those in females (not significant). The effects of either dose of DZN were statistically distinguishable from the smaller effect of CPF (*p* < 0.02 and *p* < 0.01 for comparison with 0.5 or 2 mg/kg DZN, respectively). The treatment effects in the brainstem were not significantly different from those in the fore-brain (no treatment × region interaction), so only main treatment effects are reported.

## Discussion

Earlier work in adults showed that doses of OPs high enough to elicit signs of systemic toxicity can regionally up-regulate AChE expression specifically involving the AChE-R variant, which is associated with repair mechanisms (Damodaran et al. 2003; Kaufer et al. 1998; Perrier et al. 2005; Sternfeld et al. 2000), thus mimicking the sequelae of stress (Meshorer et al. 2002) and head injury (Shohami et al. 2000). The present results in developing neuronotypic cells *in vitro* and in neonatal rat brain regions *in vivo* indicate that, during development, exposures to OPs instead elicit a pattern associated with progressive neurotoxicity, namely co-induction of both AChE-R and AChE-S (Cohen et al. 2002; Perrier et al. 2005; Shohami et al. 2000; Sternfeld et al. 2000); more specifically, our *in vivo* findings indicate that this pattern emerges in the developing brain even with lower, nonsymptomatic exposures. Equally important, our results support the idea that the increases in AChE expression—especially that of AChE-S, the critical factor that determines the balance between repair and neurotoxicity—are unrelated to the ability of the OPs to inhibit catalytic activity. First, in differentiating PC12 cells, 30 μM CPF, DZN, or CPO all produced equivalent increases in the expression of the AChE-R variant, despite the fact that CPO is orders of magnitude more potent in inhibiting catalytic activity (Das and Barone 1999); lowering CPO to 30 nM, the biologically equivalent concentration for comparison with CPF and DZN, lowered the AChE-inductive response below that of the other OPs. Second, and even more critically, whereas CPF was as effective and DZN more effective in inducing AChE-S as it was for AChE-R, CPO failed to evoke any AChE-S increase whatsoever. Third, we found virtually identical patterns after neonatal rats were exposed *in vivo* to DZN regimens that evoke only minimal cholinesterase inhibition and no systemic signs of its attendant biologic effect, namely cholinergic hyperstimulation; as was true for the PC12 cells, CPF was less effective than DZN.

Although the majority of work on the developmental neurotoxicity of OPs has centered around the effects of CPF (Pope 1999; Slotkin 1999, 2004, 2005), recent evidence implicates a similar spectrum of actions for DZN (Abu-Qare and Abou-Donia 2001; Aluigi et al. 2005; Axelrad et al. 2002; Paraoanu et al. 2006; Qiao et al. 2001; Slotkin et al. 2006a, 2006b). Our results for effects on AChE variants in PC12 cells reinforce the similarity between CPF and DZN but also point to an important potential difference: at equimolar concentrations, DZN had a greater effect and, specifically, a greater preferential up-regulation of AChE-S. Although DZN may produce more persistent inhibition of AChE catalytic activity in the mature organism (Dembele et al. 2000), our results in the *in vivo* study instead point to other mechanisms that are responsible for induction of the specific AChE isoforms in the developing brain. Treatment of neonatal rats with 1 mg/kg CPF produces the same degree of AChE inhibition as 2 mg/kg DZN, about 10–20% (Slotkin et al. 2006b; Song et al. 1997), well below the 70% threshold for the emergence of any symptoms of intoxication (Clegg and van Gemert 1999). Nevertheless, as shown here, CPF had only marginal effects on expression of AChE variants, whereas DZN caused significant induction of both AChE-R and AChE-S; again, as in the *in vitro* study, DZN elicited the “neurotoxic” pattern, coordinate up-regulation of both the read-through and synaptic variants (Cohen et al. 2002; Perrier et al. 2005; Shohami et al. 2000; Sternfeld et al. 2000). As further evidence of the disconnection between inhibition of AChE catalytic activity and the induction of AChE transcripts, the higher dose of DZN used here reduces AChE enzymatic activity preferentially in females (Slotkin et al. 2006b), whereas the effects on mRNAs encoding AChE-R and AChE-S were greater in males. Even more to the point, reducing the dose of DZN to 0.5 mg/kg produced virtually an equivalent effect on AChE isoform expression, despite the fact that there is no inhibition of catalytic activity whatsoever at the lower dose (Slotkin et al. 2006b). Our results thus suggest that, although CPF and DZN share the ability to produce developmental neurotoxicity at doses below the threshold for overt symptoms of exposure, or even for inhibition of AChE catalytic activity, DZN is far more likely to activate neurotoxic events linked to the up-regulation of AChE-S. We therefore anticipate that, because of this additional component, DZN may very well be more neurotoxic than CPF in the developing brain. This prediction is supported by two recent articles from our group (Slotkin et al. 2006a, 2006b) and is currently being explored using microarray techniques to detail the spectrum of gene families involved in the comparative developmental neurotoxicity of CPF and DZN. In any case, our findings point to the necessity of examining gene splice variants that may be important in the mechanisms or outcomes of OP-induced developmental neurotoxicity, and not just the total activity of the protein product.

The surprising dissimilarities between CPF and CPO point to potential differences in the mechanisms underlying their actions on AChE catalytic activity versus gene expression. CPO covalently binds with a reactive serine in the AChE catalytic site, producing irreversible inactivation. CPF or DZN, on the other hand, do not form covalent bonds and thus reversibly inhibit AChE by steric interaction with the same site. Our finding that CPF and DZN induce both AChE isoforms while CPO induces only the AChE-R variant is in agreement with findings from Alzheimer disease therapeutics (Darreh-Shori et al. 2004). In their study, Darreh-Shori et al. (2004) found that the reversible AChE inhibitor, tacrine, induced both AChE isoforms, whereas the essentially irreversible inhibitor, rivastigmine, mildly induced only the AChE-R isoform. There is some evidence that the relevant interactions actually involve a peripheral anionic site on the molecule rather than the catalytic center. Ligand binding to the peripheral site can block access of substrates or allosterically alter the catalytic efficiency of AChE, but more importantly for our considerations, it interferes with its nonenzymatic adhesion functions that are required for axonogenesis and other developmental events (Bourne et al. 2003; Johnson and Moore 2003; Kousba et al. 2004; Sentjurc et al. 1999). Although the OP oxons have been explored for interactions with the peripheral anionic site, our findings suggest the potential importance of a comparative examination of native OPs versus their oxons to determine the extent to which this target participates in developmental neurotoxicity. We thus anticipate that the induction of AChE variants associated with neurotoxicity may require a different set of molecular interactions from those elicited by covalent binding of OP oxons to the catalytic site of AChE.

Finally, if the expression pattern of AChE variants plays a role in the developmental neurotoxicity of OPs, then, based on our findings, it would be expected that males would be affected to a greater extent by the DZN *in vivo* treatment regimens examined here. Indeed, this prediction is consistent with earlier results for effects of CPF on neuronal structural proteins (Garcia et al. 2003), for long-term changes in central and peripheral nervous system synaptic function (Aldridge et al. 2004; Meyer et al. 2004), for structural abnormalities such as cortical thinning (Byers et al. 2005), for tests of cognitive performance (Aldridge et al. 2005; Levin et al. 2002), and for locomotor activity (Dam et al. 2000). For DZN, given the additional participation of sex-selective effects on AChE-S, we would anticipate even stronger sex-selectivity of neurotoxic outcomes. Perhaps just as critically, even in the control group we found substantially higher expression of AChE-S in males than in females. This type of underlying difference in the “neurotoxic” variant of AChE may thus contribute to greater vulnerability of neonatal males to neurotoxicant injury in general and not just to the OP pesticides. In sum, our results support the idea that non-enzymatic functions of AChE splice variants are involved in the mechanisms for the developmental neurotoxicity of OPs, and that the various OPs differ in the degree to which they recruit this mechanism.

## Figures and Tables

**Figure 1:**
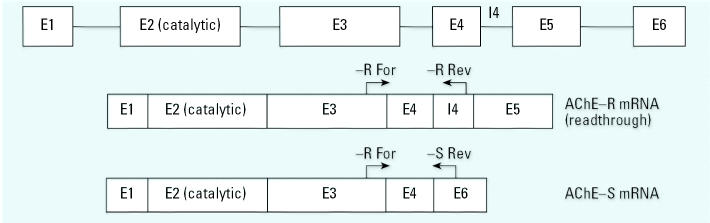
AChE isoforms resulting from alternative splicing of the AChE pre-mRNA. Abbreviations: E, exon; For, forward; I, intron; Rev, reverse. The AChE-R variant mRNA is the product of splicing and includes intron (I) 4 and omits exon (E) 6. AChE-S omits intron 4 and extends through exon 6. Arrows represent primers used in PCR detection of each splice variant.

**Table 1:** Primers and conditions for RT-PCR analysis of AChE splice variants.

AChE variant	Primers	Product length	Cycles for PC12 samples	Cycles for brain samples
AChE-R	Forward: 5′-CCCTCACTGAACTACACCGTGGAG-3′ Reverse: 5′-GTCCTTCCAACCCTTGCCGCCTTG-3′	327 bp	29	34
AChE-S	Forward: 5′-CCCTCACTGAACTACACCGTGGAG-3′ Reverse: 5′-CGGCCTTCCACTGGCGCTCC-3′	310 bp	24	29
